# Mechanical Stimulation of the HT7 Acupuncture Point to Reduce Ethanol Self-Administration in Rats

**DOI:** 10.1155/2017/6578621

**Published:** 2017-05-30

**Authors:** Suk-Yun Kang, O Sang Kwon, Ji-Young Moon, Seong Jin Cho, Kwang-Ho Choi, Junbeom Kim, Seong-Hun Ahn, Yeonhee Ryu

**Affiliations:** ^1^KM Fundamental Research Division, Korea Institute of Oriental Medicine, Daejeon 34054, Republic of Korea; ^2^Department of Meridians & Acupoints, College of Oriental Medicine, Wonkwang University, Iksan 54538, Republic of Korea

## Abstract

**Background:**

Alcoholism, which is a disabling addiction disorder, is a major public health problem worldwide. The present study was designed to determine whether the application of acupuncture at the Shenmen (HT7) point suppresses voluntary alcohol consumption in addicted rats and whether this suppressive effect is potentiated by the administration of naltrexone.

**Methods:**

Rats were initially trained to self-administer a sucrose solution by operating a lever. A mechanical acupuncture instrument (MAI) for objective mechanical stimulation was used on rats whose baseline response had been determined. In addition, the effect of HT7 acupuncture on beta-endorphin concentration and ethanol intake via naltrexone were investigated in different groups.

**Results:**

We found that ethanol intake and beta-endorphin level in rats being treated with the MAI at the HT7 point reduced significantly. The treatment of naltrexone at high doses reduced the ethanol intake and low-dose injection of naltrexone in conjunction with the MAI also suppressed ethanol intake.

**Conclusions:**

The results of the current study indicate that using the MAI at the HT7 point effectively reduces ethanol consumption in rats. Furthermore, the coadministration of the MAI and a low dose of naltrexone can produce some more potent reducing effect of ethanol intake than can acupuncture alone.

## 1. Introduction

Alcohol addiction is a serious public health problem with significant social and economic consequences worldwide. There are only three US Food and Drug Administration- (FDA-) cleared drugs for the treatment of alcohol abuse (naltrexone, acamprosate, and disulfiram) [1]. Naltrexone, a mu-opioid receptor antagonist, was cleared by the US FDA for the treatment of alcoholism in 1994. Many studies have demonstrated that alcohol addiction is related to endogenous opioid systems [2], and antagonizing with mu-opioid receptors is effective in decreasing alcohol consumption in heavy drinkers and in treating alcohol addiction [3, 4]. However, naltrexone is still far from perfect in treating all cases of alcoholism and side effects, including nausea and hepatotoxicity, confound treatment in patients with liver disease [5].

Therefore, better therapies are needed. As the best-known alternative medicine treatment, acupuncture therapies, including electroacupuncture, manual acupuncture, and chemical acupuncture, are commonly used to treat drug abuse in Eastern countries [6, 7]. Although the biological mechanisms of acupuncture are not clear, acupuncture treatment can counteract the functional hyperactivity and hypoactivity of neurotransmitter systems caused by external stimuli and can simultaneously contribute to the maintenance of a biochemical balance and homeostasis in the body [8, 9]. The benefit of acupuncture is that it has the potential to help drug abusers stay away from drugs without major adverse side effects.

In particular, the HT7 (*Shenmen*) acupuncture point is the source point of the heart meridian and has frequently been used to treat mental disorders, including drug addiction, anxiety, and depression [10, 11]. Previous clinical and preclinical studies have shown that stimulation of the HT7 acupuncture point improves ethanol-induced anxiety and ethanol intake via the modulation of the corticotropin-releasing factor and neuropeptide Y in the amygdala [12] and the GABA neuron in the ventral tegmental area (VTA) [13]. In addition, repeated electroacupuncture to ST36 (*Joksamni*) acupuncture point effectively decreases the consumption of and preference for ethanol without resulting in a rebound increase in ethanol intake when the treatment is terminated [14].

Based on the above-mentioned studies, we hypothesized that acupuncture therapy would reduce the ethanol consumption and the coadministration of naltrexone and acupuncture could regulate the suppressive effect of alcohol intake in ethanol self-administration. Therefore, the present study was designed to examine the following: (1) whether mechanical acupuncture to the acupuncture point would decrease alcohol intake and plasma beta-endorphin concentration compared to controls that did not receive any treatment; (2) whether naltrexone would have an effect on alcohol intake, dose-dependently; and (3) whether mechanical acupuncture treatment could be affected by the injection of a lower dose of naltrexone in alcohol-addicted rats.

## 2. Materials and Methods

### 2.1. Animal Preparation

The animals used in this study were male Wistar rats weighing 250 to 280 g, provided by Orient Bio (Sungnam, Korea). The rats were housed in colony cages with free access to food and water and maintained on a 12 h light, 12 h dark cycle throughout the study period. All the procedures used in the present study were reviewed and approved by the Animal Care and Use Committee at the Korea Institute of Oriental Medicine (KIOM, Daejeon, Korea) with reference number #15-066 and conformed to NIH guidelines (NIH publication number 86-23, revised 1985). Every effort was made to minimize animal distress and discomfort and to reduce the number of animals used in the study.

### 2.2. Ethanol Self-Administration

Ethanol self-administration was performed in an operant chamber (measuring 31.8 cm × 25.4 cm × 26.7 cm, MED Associates Inc., Georgia, VT, USA), equipped with two response levers and a house light. When the experimental animals press the active lever once, a 0.1 mL drop of 10% ethanol solution was dispensed onto a dish kept on the center panel of the operant chamber, and the light was turned on during the self-administration session. On the other hand, responses on the inactive lever were recorded but had no significant consequences. The animals underwent behavioral training five days a week, for 30 minutes a day. Using the modified sucrose-fading method described previously [15]; they were trained to self-administer ethanol orally. Briefly, the rats gradually became accustomed to the behavior during the training period (to advance the process of approaching the active lever and the dish). To facilitate the essential process of pressing the lever, 20% sucrose solution was initially used to train all the animals for self-administration. After the animals learned to push the active lever firmly in order to keep drinking the 20% sucrose solution, 10% sucrose solution was used for the next step of active lever response. When a stable baseline reaction was established, concentration of the sucrose solution was gradually reduced to 0%, and ethanol concentration was ultimately increased to 10%. The detailed step was as listed below: 1 week with 2% ethanol in 10% sucrose, 1 week with 5% ethanol in 10% sucrose, and next 1 week with 10% ethanol in 5% sucrose. Finally, 10% ethanol alone was presented as the addiction reinforcer. After rats showed a stable response for the 10% ethanol solution and met an established criterion for ethanol baseline response, behavioral testing was initiated. The criteria for the ethanol baseline were determined by the mean value of three consecutive ethanol intakes, using the animals that showed a stable response to 10% ethanol, with a variation of less than 20%. The total number of ethanol intake sessions was carried out for each treatment group over 3 days, during which the induced alcohol intake was stable, and the amount of ethanol intake was averaged. The results of ethanol intake in each experimental animal were expressed as a percent of active lever response frequency, comparing acupuncture or drug treatment before and after.

### 2.3. Acupuncture Treatment

Traditional manual acupuncture is the act of inserting a needle into the acupuncture point, sometimes twisting the needle. To mimic the vibrations produced by manual acupuncture stimulation, a mechanical acupuncture instrument (MAI) was developed by researchers at Daegu Haany University and the Korea Institute of Oriental Medicine in South Korea [16]. Unlike conventional acupuncture methods, this device consisted of a custom-made control unit and a cell phone vibrator connected to an acupuncture needle. The MAI was arranged at 1.3 m/sec^2^ in intensity and 85 Hz in frequency for experiments. All rats were habituated to the experimental procedures, which included handling and acupuncture manipulation without needle insertion for at least 1 week before the study. Stainless-steel needles (0.18 mm diameter and 20 mm length) were inserted vertically to a depth of 3 mm into acupuncture points and the tail of rats lightly restrained by hands. The MAI was applied bilaterally at the acupuncture points and the tail for 30 seconds and maintained for up to 1 min after needle insertion. The experimental animals in the HT7 and ST36 treatment group were administrated at the HT7 on the forelimb, and the ST36 on the lateral side of the tibia. The HT7 acupuncture point was located on the transverse crease of the wrist of the forepaw, radial to the tendon of the flexor carpi ulnaris muscle, and ST36 point was situated in the lateral side of the stifle joint adjacent to the anterior tubercle of the tibia approximately 3 mm below and lateral to the midpoint of the knee. The anatomical location of the acupuncture points stimulated in the rats corresponded to the acupuncture points in the animal acupuncture atlas [17]. The rat's tail (1/3 tail length from the proximal region) was used as a stimulation control site to determine the effect of mechanical stimulation at nonacupuncture points. All acupuncture treatments were provided by a single skilled acupuncturist.

### 2.4. Plasma Beta-Endorphin Assays

To assay plasma levels of beta-endorphin concentration, the animals were anesthetized using isoflurane and decapitated after alcohol intake test, and 1 ml of blood was collected in tubes containing EDTA (1.6 mg/ml). The blood samples were centrifuged at 3,000*g* for 10 minutes at 4°C, and the plasma was separated for measuring beta-endorphin concentration. The beta-endorphin levels in the plasma were measured using the commercial ELISA kit (MSB733830, MyBioSource, San Diego, CA, USA). The values are expressed in ng/ml.

### 2.5. Drug Preparation and Treatment

The effects of naltrexone were investigated in different groups of rats using the same ethanol self-administration procedures. Before use, naltrexone was dissolved in saline (0.9% NaCl) at concentrations of 0.1, 0.3, 1.0, and 3.0 mg/kg. Each drug was administered intraperitoneally at a volume of 100 *μ*L, and rats were pretreated 30 minutes before the ethanol self-administration.

### 2.6. Statistical Analysis

All data are expressed as the mean ± standard error of mean (SEM). Statistical analyses were performed using Prism 6 (GraphPad Software, San Diego, CA, USA). For the ethanol intake analysis, data were compared using a one-way ANOVA. Post hoc analysis was performed using Tukey's multiple comparison test in order to determine *P* value among experiment groups. A value of *P* < 0.05 was considered to be statistically significant.

## 3. Results

### 3.1. Effect of Mechanical Acupuncture Treatment on Ethanol Self-Administration

**Figure 1:**
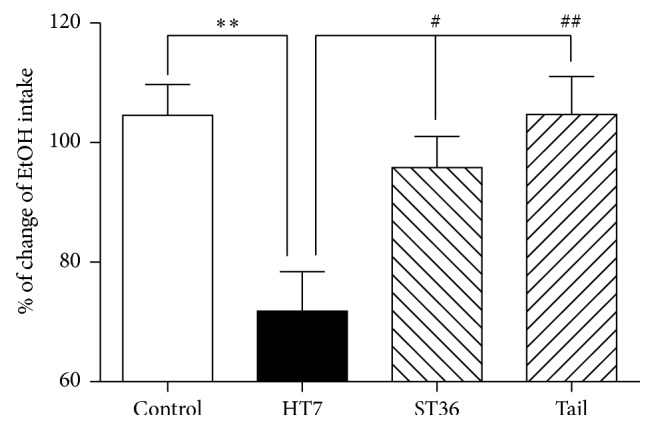
The effects of mechanical acupuncture on ethanol intake in alcohol-addicted rats. The administration of mechanical acupuncture to the HT7 acupuncture point (HT7 group), but not to the ST36 point or the tail (ST36 group and tail group), significantly decreased the ethanol intake compared to the control animals (control group). The ethanol intake in the ST36 and tail group increased compared to the HT7 group. The results are shown as the mean ± SEM of the ethanol consumption in each group. Control group, *n* = 6; HT7 group, *n* = 6; ST36 group, *n* = 6; tail group, *n* = 6; Student's *t*-test. ^*∗∗*^*P* < 0.01, control group versus HT7 group. ^#^*P* < 0.05 and ^##^*P* < 0.01, HT7 group versus ST36 group and tail group.

We first tested whether the administration of mechanical acupuncture to the HT7 and ST36 points and the tail had a suppressive effect on ethanol intake when using the self-administration operant chamber in the alcohol-addicted rats. The administration of mechanical acupuncture to the HT7 acupuncture point, but not to the ST36 point or the tail, significantly decreased the ethanol consumption compared with the control animals (^*∗∗*^*P* < 0.01; control: 104.6 ± 12.7%, *n* = 6 versus HT7: 71.8 ± 16.1%, *n* = 6). The mechanical acupuncture to the ST36 point and the tail did not decrease the ethanol consumption (ST36: 94.2 ± 10.6%, *n* = 6 and tail: 104.7 ± 15.6%, *n* = 6; Figure 1) compared to the control animals. There was also an increase in ethanol consumption in the ST36 point and the tail group compared to the HT7 groups, respectively (^#^*P* < 0.05 and ^##^*P* < 0.01).

### 3.2. Effect of Mechanical Acupuncture Treatment on Plasma Beta-Endorphin Concentration

**Figure 2:**
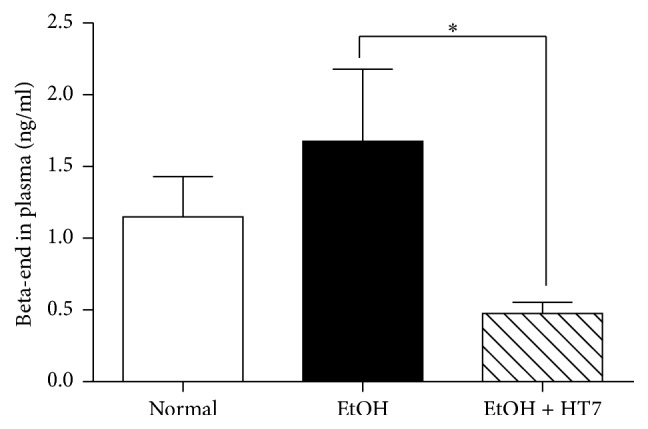
The effects of mechanical acupuncture on plasma beta-endorphin in alcohol-addicted rats. The rats with alcohol addiction (EtOH group) did not affect plasma beta-endorphin level compared with normal animals group (normal group). The administration of mechanical acupuncture to the HT7 acupuncture point (EtOH + HT7 group) significantly decreased the plasma beta-endorphin level compared to the control animals (EtOH group). The results are shown as the mean ± SEM of the plasma beta-endorphin level in each group. Normal group, *n* = 4; EtOH group, *n* = 4; EtOH + HT7 group, *n* = 4; Student's *t*-test. ^*∗*^*P* < 0.05, EtOH group versus EtOH + HT7 group.

Next, we measured the level of beta-endorphin, an endogenous opioid neuropeptide, in plasma using ELISA kits. As shown in Figure 2, the basic plasma beta-endorphin level in normal rats (normal) was 1.14 ± 0.48 ng/ml. In the group of rats with alcohol addiction (EtOH), the levels of this peptide increased to 1.68 ± 0.85 ng/ml. Level of beta-endorphin observed in rats treated with mechanical acupuncture into the HT7 acupuncture point (EtOH+HT7) was 0.48 ± 0.19 ng/ml. No significant differences were observed in alcohol-addicted animals in comparison to the corresponding group with normal animals (no significance, but showed a tendency toward increasing; normal, *n* = 4 versus EtOH, *n* = 4). On the other hand, the plasma beta-endorphin level in the group treated with mechanical acupuncture into the HT7 acupuncture point was statistically different from the group with alcohol addiction (^*∗*^*P* < 0.05; EtOH versus EtOH + HT7, *n* = 4).

### 3.3. Effect of Naltrexone Injection on Ethanol Self-Administration

**Figure 3:**
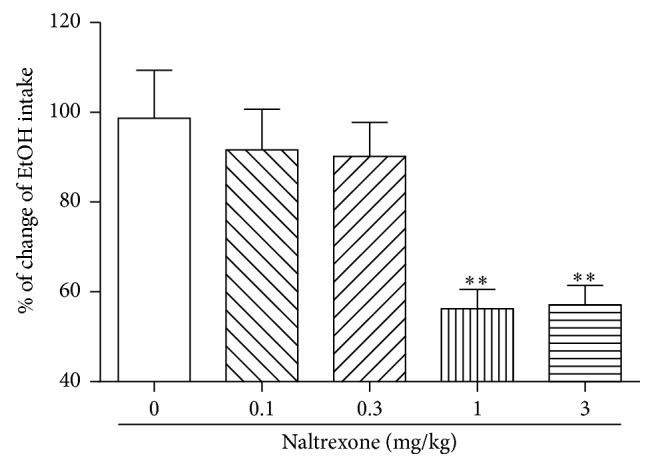
The effects of a naltrexone injection on ethanol intake in the alcohol-addicted rats. Though treatment with low doses (naltrexone 0.1 and 0.3 mg/kg groups) of naltrexone did not affect ethanol intake, treatment with higher doses (naltrexone 1 and 3 mg/kg groups) of naltrexone significantly decreased the ethanol consumption compared with the vehicle-treated control group (naltrexone 0 mg/kg group). The results are shown as the mean ± SEM of the ethanol consumption in each group. Naltrexone 0 mg/kg group, *n* = 7; naltrexone 0.1 mg/kg group, *n* = 7; naltrexone 0.3 mg/kg group, *n* = 7; naltrexone 1 mg/kg group, *n* = 7; naltrexone 3 mg/kg group, *n* = 7; Student's *t*-test. ^*∗∗*^*P* < 0.01, naltrexone 0 mg/kg group versus Naltrexone 1 and 3 mg/kg group.

We evaluated the dose-dependent effect of a naltrexone injection on ethanol intake. As shown in Figure 3, the administration of low doses (0.1 or 0.3 mg/kg) of naltrexone did not have any significant suppressive effects on ethanol intake compared with the vehicle-treated control group (0.1 mg/kg: 91.7 ± 24.0%, *n* = 7 and 0.3 mg/kg: 90.2 ± 20.0%, *n* = 7). However, the administration of higher doses (1 or 3 mg/kg) of naltrexone significantly decreased the amount of ethanol consumption compared to the control animals (^*∗∗*^*P* < 0.01; 0 mg/kg: 98.7 ± 28.3%, *n* = 7 versus 1 mg/kg: 56.2 ± 11.5%, *n* = 7 and 3 mg/kg: 57.1 ± 11.5%, *n* = 7).

### 3.4. Effect of Coadministration of Naltrexone with Mechanical Acupuncture on Ethanol Self-Administration

We next examined whether the administration of naltrexone, alone or in combination with mechanical acupuncture treatment, had any effect on ethanol consumption.

The administration of mechanical acupuncture to the HT7 point significantly decreased ethanol intake compared to that of the control group (^*∗*^*P* < 0.05; control: 107.9 ± 11.7%, *n* = 7 versus 0 mg/kg + HT7: 81.6 ± 11.9%, *n* = 7). The systemic administration of low doses (0.1 or 0.3 mg/kg) of naltrexone did not change the ethanol consumption compared to the control animals. However, when a low dose of naltrexone was coadministered with mechanical acupuncture, it significantly suppressed ethanol intake compared with the same dose of naltrexone alone (^*∗∗*^*P* < 0.01; 0.1 mg/kg: 91.6 ± 23.9%, *n* = 7 versus 0.1 mg/kg + HT7: 67.1 ± 13.4%, *n* = 7, ^*∗∗∗*^*P* < 0.001; 0.3 mg/kg: 90.1 ± 20.0%, *n* = 7 versus 0.3 mg/kg + HT7: 54.8 ± 8.9%, *n* = 7). In particular, the coadministration of 0.3 mg/kg of naltrexone and acupuncture significantly decreased the amount of ethanol consumption compared to the mechanical acupuncture animals (^#^*P* < 0.05).

## 4. Discussion

The advantage of acupuncture therapy is that it has the potential to treat a significant number of drug abusers without major adverse side effects. Acupuncture is mainly used to produce an analgesic effect and to treat inflammation and drug abuse in Eastern countries [6, 7, 18, 19]. Acupuncture can concurrently treat the functional overactivity and underactivity of neurotransmitter systems caused by noxious stimuli [8]. Previous experimental results in our laboratory have provided scientific support for this alternative treatment approach by demonstrating that the subcutaneous treatment of bee venom produces a robust antinociceptive effect in various animal models of pain, including the formalin test [20], the chronic constriction injury model [21], and several models of arthritis [19]. Recently, several investigators have reported on studies of acupuncture and electroacupuncture worldwide for the treatment of some mental disorders, including drug abuse, anxiety, and withdrawal syndrome, which are derived from the dysregulation of specific neurochemical elements involved in reward and stress in the brain [6, 12, 22]. Because the HT7 and ST36 points are the most frequently used acupuncture points to treat drug abuse, in the present study, we investigated the importance of these acupuncture points in ethanol self-administration. The present study demonstrated that stimulation of the HT7 acupuncture point using a mechanical acupuncture instrument, but not the ST36 point or a nonacupuncture point, significantly decreased ethanol self-administration, suggesting that this effect is specific to the HT7 acupuncture point (Figure 1). The mechanical acupuncture instrument has the advantage of a shorter stimulus time (30 sec) than that of typical electroacupuncture, which is approximately 30 min [16], and minimal handling and restraint decrease stressful behaviors, such as avoidance and vocalization, during acupuncture.

**Figure 4:**
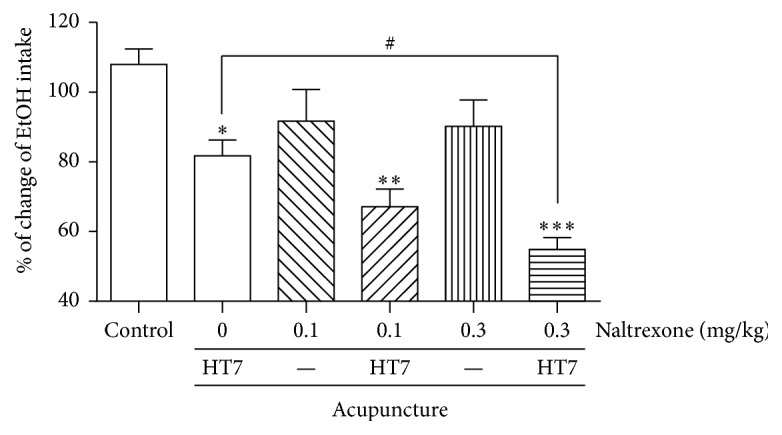
The effects of naltrexone in combination with mechanical acupuncture on the ethanol intake in the alcohol-addicted rats. The administration of mechanical acupuncture to the HT7 point (naltrexone 0 mg/kg + HT7 group) significantly decreased ethanol intake compared to the control animals (control group, ^*∗*^*P* < 0.05, control group versus Naltrexone 0 mg/kg + HT7 group). In addition, a combination therapy of a low dose of naltrexone and mechanical acupuncture (naltrexone 0.1 and 0.3 mg/kg + HT7 groups) significantly suppressed the ethanol intake compared with the same dose of naltrexone alone (naltrexone 0.1 and 0.3 mg/kg groups, ^*∗*^*P* < 0.05 and ^*∗∗*^*P* < 0.01, naltrexone 0.1 and 0.3 mg/kg group versus naltrexone 0.1 and 0.3 mg/kg + HT7 group). In particular, the coadministration of 0.3 mg/kg of naltrexone and acupuncture (naltrexone 0.3 mg/kg + HT7 group) significantly decreased the ethanol intake compared to the mechanical acupuncture group (^#^*P* < 0.05, naltrexone 0.3 mg/kg + HT7 group versus naltrexone 0 mg/kg + HT7 group). The results are shown as the mean ± SEM of the ethanol consumption in each group. Control group, *n* = 7; naltrexone 0 mg/kg + HT7 group, *n* = 7; naltrexone 0.1 mg/kg group, *n* = 7; naltrexone 0.1 mg/kg + HT7 group, *n* = 7; naltrexone 0.3 mg/kg group, *n* = 7; naltrexone 0.3 mg/kg + HT7 group, *n* = 7; Student's *t*-test.

Next, we demonstrated that administration of mechanical acupuncture into the HT7 acupuncture point statistically decreased the beta-endorphin, an endogenous opioid neuropeptide, concentration in the plasma. Beta-endorphin is known to play a key role in a mesolimbic reward system. Hence, it has been suggested that mechanical acupuncture has a modulating effect on the endogenous opioid system in alcohol addiction. In addition, the findings of this study demonstrated that low doses of naltrexone had no effect on ethanol consumption, but higher doses significantly suppressed ethanol consumption compared to control animals (Figure 3). Moreover, the coadministration of mechanical acupuncture to the HT7 point and a low dose of naltrexone had some more suppressive effect of alcohol intake (Figure 4). As FDA-approved drug, naltrexone used the most common drug for the treatment of alcohol abuse can decrease ethanol self-administration, suggesting that endogenous opioid systems contribute to ethanol reward in alcohol-addicted rats [23]. Many researchers have proposed that blacking of endogenous opioid system is very effective for treating alcoholism [24]. Acupuncture related to the release of endogenous opioid peptides (enkephalin, beta-endorphin and dynorphin) from the central nervous system, and opioids are known to increase dopaminergic neuron activity in the mesolimbic brain region [7, 8]. Acute withdrawal from chronic ethanol intake may contribute to the reduced release of endogenous opioid peptides and the excitability of dopaminergic neurons in the VTA [25]. These results are consistent with the suppressive effect of naltrexone on alcohol intake observed in the present study. Although predicting which patients suffering from alcohol addition will completely recover is impossible, our results demonstrate that a low dose of naltrexone in combination with mechanical acupuncture to the HT7 point significantly decreases alcohol intake compared to a control group. These results indicate that mechanical acupuncture enhances the alcohol addiction treatment effect of naltrexone. Collectively, these findings, including the present results, indicate that mechanical acupuncture therapy can have a powerful suppressive effect on alcohol intake in ethanol self-administration. In conclusion, the present study demonstrates that the effect of mechanical acupuncture to the HT7 point combined with a low dose of naltrexone can reduce the alcohol intake response in alcohol addiction. These results suggest that the clinical use of mechanical acupuncture treatment can be a novel strategy in the early management of alcoholic patients.
